# Anti-feeding efficacy of Activyl^®^ Tick Plus topical treatment of dogs against *Phlebotomus perniciosus*

**DOI:** 10.1186/1756-3305-7-217

**Published:** 2014-05-09

**Authors:** Régis Frenais, Annie Flochlay-Sigognault, Gaëlle Milon-Harnois

**Affiliations:** 1 MSD Animal Health Innovation SAS, 7 rue Olivier de Serres, CS 67131, Beaucouzé cedex 49071, France

**Keywords:** Indoxacarb, Permethrin, Sandfly, Phlebotomus perniciosus, Anti-feeding efficacy, Knock-down effect

## Abstract

**Background:**

Topical permethrin treatment is known to prevent feeding of sandflies on dogs. This study investigated the anti-feeding efficacy and the immediate insecticidal efficacy (knock-down effect) of topical treatment of dogs with a new commercially available combination of indoxacarb and permethrin (Activyl^®^ Tick Plus), compared with a negative control.

**Methods:**

Sedated dogs were individually exposed to unfed female sandflies in a darkened chamber for one hour 2, 7, 14, 21 and 29 days after treatment. Mean fly feeding and survival rates in the two groups after one hour of exposure were used to calculate the anti-feeding efficacy and the knock-down effect, respectively.

**Results:**

On Days 2, 7, 14, 21 and 29 post treatment, the anti-feeding efficacy was 99, 98, 96, 88 and 84% based on geometric means. The mean number of fed sandflies in the treated group was significantly lower than in the control group mean at every evaluation time point. The knock-down effect, measured at one hour after exposure of the flies to treated dogs, was 32, 27, 9, 0 and 4% based on geometric means, at the same time points. The number of dead flies was significantly higher in the treated group on Days 2 and 7 post-treatment. No adverse effects of treatment were observed at any time during the study.

**Conclusions:**

Activyl^®^ Tick Plus treatment of dogs provided a high anti-feeding efficacy against *Phlebotomus perniciosus* from 2 to 21 days post treatment, with continuing significant anti-feeding to 29 days post-treatment, and was well tolerated. Some knock-down effect following one hour of exposure of flies to treated dogs was observed in the first week after treatment.

## Background

Infections in dogs with the protozoan parasite *Leishmania infantum* are widespread in southern Europe. Leishmaniases are vector-borne diseases, the promastigote stage of the parasite being transmitted to the host during the blood feeding of an insect vector, the sand fly [1]. The sandfly *Phlebotomus perniciosus* is the most significant vector for canine leishmaniasis in southern Europe [2]. *Leishmania infantum* is also a critical human health problem since dogs, as major companion animals, serve as the main reservoir [3]. Several studies showed that the prevalence of human leishmaniasis could be significantly decreased with control of leishmaniasis in dogs [4-6]. Therefore, reducing the risk of *L. infantum* infection by protecting dogs from sandfly bites has become a strategy in veterinary medicine. Podaliri Vulpiani *et al*. [5] reviewed the methods of control of the *L. infantum* dog reservoir and discussed the results of the studies conducted over the last decade that aimed at demonstrating the efficacy of topical treatment of dogs exposed to sandfly bites [5]. Topical treatment with permethrin alone [7] or in combination with pyripoxyfen [8], with imidacloprid [9] or with dinotefuran and pyriproxyfen [10] can provide anti-feeding efficacy against sandflies. A preparation combining permethrin with indoxacarb is now also commercially available for treatment of tick and flea infestations in dogs. Indoxacarb is a pro-insecticide that is bioactivated by the insect into a metabolite that is highly effective against adult and developing stages of fleas [11,12]. This compound does not show insect repellent activity [13]. This study investigated the duration and strength of the anti-feeding efficacy and the knock-down effect against *P. perniciosus* following topical treatment of dogs with a combination of indoxacarb and permethrin (Activyl^®^ Tick Plus, Merck/MSD Animal Health).

## Methods

The study protocol was approved by the ethics committee of Charles River Laboratories Preclinical Services Ireland Ltd., prior to the start of the study. Sixteen healthy previously ectoparasiticide untreated Beagle dogs (8 male and 8 female) were identified with a subcutaneous microchip and individually housed. They were weighed, acclimatized for 6 days and randomly assigned to one of two groups, blocking on gender and bodyweight, using a computer generated randomization. Dogs received a commercial diet once daily at standard feeding rates with water *ad libitum*, except during the sandfly exposure period. Male dogs were all 6 months old and weighed 8.3 kg to 10.1 kg. Female dogs were between six and eleven months of age and weighed between 7.7 kg and 11.2 kg.

On the first day of the experimental period, dogs in the treatment group received a topical spot-on application of Activyl^®^ Tick Plus, containing indoxacarb (150 mg/mL) and permethrin (480 mg/mL), at the minimum recommended dose rate of 15 mg indoxacarb and 48 mg permethrin per kg body weight. This treatment was administered as either a single spot to the skin between the shoulder blades, or as two spots (one between the shoulder blades and the other at the base of the tail) depending on the body weight of the dog. The other group of dogs received the same volume of inactive excipient solution applied in the same way. No evidence of treatment run-off was observed from any dog in either the Activyl^®^ Tick Plus group or negative control group.

**Figure 1:**
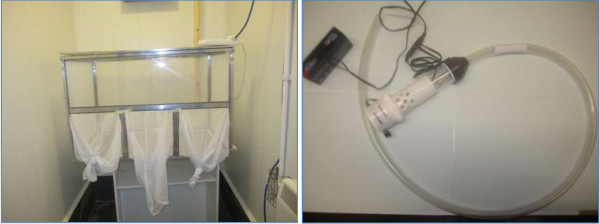
Sandfly infestation chamber and aspirator.

Two, 7, 14, 21 and 29 days after the initial treatment, each dog was anaesthetized (i.m. injection of 0.15 mL/kg body weight ketamine and 0.15 mL/kg body weight xylazine) and then individually placed in a chamber (see Figure 1) measuring approximately 0.6 m × 0.6 m × 0.9 m (length × width × height) within a separate room held at > 49% relative humidity. Treated and control groups were exposed to sandflies in separate chambers to avoid possible cross-contamination. Approximately 75 adult female, non-blood fed, 3–5 day post-hatching [14] sandflies (*P. perniciosus*) were introduced to the chamber and the room lights were then turned off for approximately one hour. A few males (10–20) were included with female flies to encourage feeding; however, males were not included in subsequent evaluation of treatment effects.

After the one-hour exposure period, dogs were treated with atipamezole hydrochloride (i.m. 0.06 mL/kg body weight) to reverse the effects of the anaesthetic agents, the room lights were turned on and live sandflies were collected from each exposure chamber into a vented, labeled container, using an aspirator. No attractant light was used to draw live sandflies away from the dog. Each dog was checked for dead or feeding flies and then removed from the chamber. All dead and moribund flies (either on the dog or in the chamber) were collected using forceps and placed into a separate container. Live sandflies were killed by freezing and all female sandflies (dead and live) were categorized as engorged (fed) or unengorged (unfed) by examination with a stereomicroscope against a white Petri dish background to detect blood meal traces. All female flies were classified into one of four categories: alive unfed, alive fed, dead unfed or dead fed. All study personnel carrying out general health observations, clinical observations, sandfly exposure, and fly counts were masked as to dog treatment status.

Anti-feeding efficacy was calculated by comparing the geometric mean numbers of fed (dead and alive) female sandflies in the treated group versus the control group, at each time point after treatment. Knock-down effect was determined based on a comparison between the two groups of the geometric mean sandfly survival rates (evaluation of alive flies after one hour of exposure to treated dogs). Anti-feeding efficacy and knock-down effect formulae are shown below:

$$\text{Anti}‒\text{feedingefficacy}=\frac{\left(\mathrm{GM}\;\text{fedC}‒\mathrm{GM}\;\text{fedT}\right)}{\mathrm{GM}\;\text{fedC}}\times 100$$

where GM fedC and GM fedT are the geometric mean numbers of fed (engorged) sandflies in the control and treated groups, respectively.

$$\text{Knock}‒\text{down}\;\text{effect}=\frac{\left(\mathrm{GM}\;\text{aliveC}‒\mathrm{GM}\;\text{aliveT}\right)}{\mathrm{GM}\;\text{aliveC}}\times 100$$

where GM aliveC and GM aliveT are the geometric mean numbers of alive (fed + unfed) sandflies in the control and treated groups, respectively.

For statistical analysis, the individual dog was the experimental unit to test the hypothesis that there were no differences between groups (two-sided tests, 5% significance level). In addition, mean fed female *P. perniciosus* and mean alive female *P. perniciosus* were transformed using log_1O_(count + 1) then formally analyzed using mixed ANOVA models for repeated measures (SAS version 9.2, SAS Institute, Cary, NC 2008).

## Results

Sandfly counts and categorization, in the control and treated groups at each time point after treatment, are presented in Table 1.

**Table 1:** Sandfly counts and categorization

**Study group**		**Alive sandfly**								**Dead sandfly**							
		**Fed**				**Unfed**				**Fed**				**Unfed**			
		**Min**	**AM**	**GM**	**Max**	**Min**	**AM**	**GM**	**Max**	**Min**	**AM**	**GM**	**Max**	**Min**	**AM**	**GM**	**Max**
Day 2	Negative control	21	43.6	41.6	60	12	27.6	24.8	52	0	0	nc	0	0	1.0	nc	3
	Activyl^®^ Tick Plus	0	0.3	nc	1	35	49.0	48.1	62	0	0.5	nc	2	4	14.5	10.7	37
Day 7	Negative control	33	46.0	45.4	56	12	22.3	20.6	41	0	0	nc	0	0	4.5	nc	9
	Activyl^®^ Tick Plus	0	0.6	nc	3	33	50.3	49.5	63	0	0.5	nc	2	4	15.1	12.7	30
Day 14	Negative control	30	46.5	44.9	63	6	20.6	18.8	30	0	0	nc	0	0	6.1	nc	18
	Activyl^®^ Tick Plus	0	3.3	nc	14	47	57.9	57.6	66	0	0.1	nc	1	1	9.3	6.6	21
Day 21	Negative control	44	53.8	53.5	62	11	13.6	13.4	19	0	0	nc	0	1	2.5	2.3	4
	Activyl^®^ Tick Plus	2	7.6	6.5	12	57	62.8	62.6	69	0	0	nc	0	1	2.5	2.3	4
Day 29	Negative control	22	38.4	37.1	47	17	27.4	26.4	38	0	0	nc	0	2	6.4	5.8	10
	Activyl^®^ Tick Plus	2	6.4	5.7	10	53	57.0	56.9	62	0	0	nc	0	1	7.5	6.2	12

AM, arithmetic mean, GM, geometric mean; nc, could not be calculated (data were transformed using log10 (count + 1) to calculate anti-feeding efficacies).

Between 54 and 76 live female sandflies and 1 to 18 dead female sandflies were recovered from negative control group dogs at each time point throughout the study, confirming the validity of the experimental infestation model.

**Figure 2:**
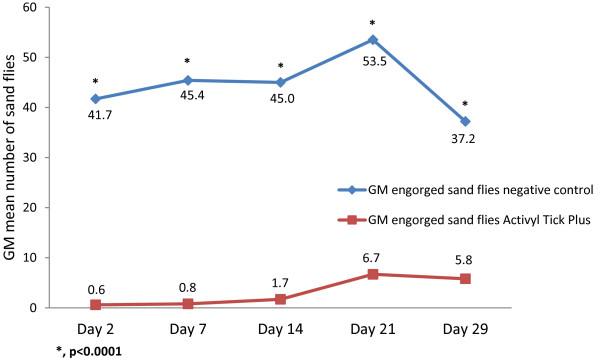
Geometric mean numbers of engorged (dead and alive) female sandflies.

The geometric mean numbers of engorged (dead and alive) female sandflies in the Activyl^®^ Tick Plus treated group versus the negative control group, at each time point after treatment, is shown in Figure 2. Compared to the negative control group, the number of unfed sandflies was significantly lower in the Activyl^®^ Tick Plus treated group at every time point (*P* < 0.0001) post treatment. The anti-feeding efficacy of > 95% was observed from 2 to 14 days post treatment, and significant anti-feeding effect of > 80% was still reported 29 days post-treatment (Table 2).

**Table 2:** Anti-feeding efficacy and knock-down effect of topical indoxacarb and permethrin treatment of dogs against sandflies (*P. perniciosus*)

	**Day 2**	**Day 7**	**Day 14**	**Day 21**	**Day 29**
Anti-feeding efficacy	99%	98%	96%	88%	84%
Knock-down effect	32%	27%	9%	0	4%

**Figure 3:**
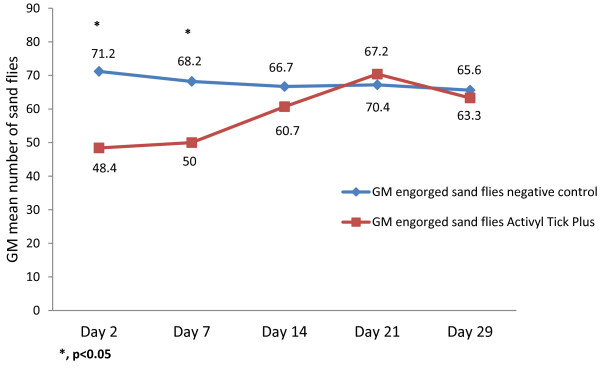
Geometric mean numbers of alive (fed and unfed) female sandflies.

The geometric mean numbers of alive (fed and unfed) female sandflies in the Activyl^®^ Tick Plus treated group versus the negative control group, at each time point after treatment, is shown in Figure 3. The number of alive sandflies was significantly lower on Days 2 and 7 (*P* < 0.05). A short-term insecticidal efficacy of > 25% was reported over one week post treatment (Table 2), but not afterwards (< 10% up to 29 days post-treatment).

No local or general adverse events were observed in either the Activyl^®^ Tick Plus treated group or the negative control group during the study.

## Discussion

Topical treatment with a commercial formulation containing permethrin and indoxacarb (Activyl^®^ Tick Plus) provided significant anti-feeding efficacy against the sandfly *P. perniciosus* for up to four weeks after application. The anti-feeding efficacy of > 95% was observed from 2 to 14 days post treatment, with significant anti-feeding effect continuing to 29 days post-treatment. This study confirmed that a topical permethrin and indoxacarb combination treatment could aid in the prevention of sandfly infestation in dogs. Although the frequency of subsequent applications was not determined in this study, the results suggest that treatment would need to be re-applied two to three weeks after initial application to maintain appropriate efficacy and prevent blood feeding, which is consistent with the duration of effects approved for similar permethrin-containing products. Advantix^®^ (Bayer), a combination of imidacloprid and permethrin, is licenced in Europe with an efficacy claim of 2 to 3 weeks against *P. papatasi* and *P. perniciosus*[9]. More recently Vectra 3D™ (Ceva), a combination of dinotefuran, permethrin and pyriproxyfen, has been shown to provide an anti-feeding efficacy of > 95% up to 14 days post treatment, with significant anti-feeding effect of > 80% continuing to 28 days post-treatment, compared with the control group [10].

The short-term insecticidal efficacy (knock-down effect) was significant over one week post treatment, but as expected no persistent immediate insecticidal activity could be evidenced afterwards. The low knock-down effect reported is consistent with the results observed with other permethrin-containing products in studies where the percent of alive sand flies was also evaluated after one hour of exposure [7,9]. In this study, the number of dead flies was not evaluated 24 hours after insecticide exposure. For this reason, no conclusion can be drawn on the insecticidal activity according to international standards [15]. However, it remains widely recognized that the anti-feeding effect of permethrin, more than its immediate insecticidal potential, is really the true benefit against transmission of *L. infantum* by phlebotomine vectors.

Prophylactic measures to control the spread of *L. infantum* have been initiated over the past 15 years through studies that aimed at demonstrating the efficacy of topical treatment of dogs exposed to sandfly bites. Collars, spot-on and spray formulations, containing pyrethroids at various concentrations, have demonstrated both anti-feeding efficacy (repulsive effect of the insecticide resulting in a decreased blood feeding) and insecticide effect (persistent absorption of the insecticide by the insect at toxic doses) [5]. The synergic repulsive/toxic action of these insecticides allows both the prevention of sandfly bites and their elimination or reduction, thereby contributing to the prevention of *L. infantum* transmission. It is widely accepted that collars have longer anti-feeding and toxicological activities (up to 6 months) compared with sprays and spot-on which have to be administered at least once a month [3]. Conversely, the onset of action of the insecticide is immediate with sprays and ranges between 24–48 hours with spot-on formulations, but the full protective activity is achieved within one week with collars, due to a slower release of the active [3]. Data from the literature have shown that the 24-hour post treatment insecticidal effect against *P. perniciosus* is comparable with deltamethrin 4% collars (25-64%) and permethrin 50%-imidacloprid 10% or permethrin 65% spot on formulations (49-67%). A short-term knock-down effect of 7% was reported with a permethrin 1.9%-pyriproxyfen 0.02% spray. Conversely, the anti-feeding effect seems higher with a permethrin 50%-imidacloprid 10% or permethrin 65% spot on (89-98%) compared to a deltamethrin 4% collar (72-90%) or a permethrin 1.9%-pyriproxyfen 0.02% spray (71%) [3,5].

Previous studies of permethrin sandfly anti-feeding efficacy, either as the single active ingredient or in various combination spot-on formulations [7-9], have shown a similar pattern of extended blood feeding prevention over two to four weeks after treatment, together with relatively shorter and lower knock-down effect. Molina *et al*. [7], particularly, demonstrated that 65% permethrin applied to dogs as a spot-on had satisfactory anti-feeding effect (> 65%) lasting 3 weeks and immediate insecticidal effects (> 40%) lasting 2 weeks after initial application, which is consistent with the present results [7]. Similarly, highly comparable results were reported by Miró *et al*. [9] with a combination of imidacloprid 10% (w/v)/permethrin 50% (w/v) spot-on, with an immediate insecticidal effect (assessed after 1 hour of sand fly exposure) within the first week of application (> 40%), and an anti-feeding effect of over 90% during the first 3 weeks of the study [9]. Recent results obtained with the addition of 4.95% dinotefuran in the combination 36.08% permethrin-0.44% pyriproxyfen have demonstrated both persistent knock-down effect and insecticidal activity 7 days (> 95%), 2 weeks (> 70%) and up to 4 weeks after treatment (about 40%) [10].

Permethrin uptake on contact by arthropods is the major route of the pharmacodynamic effect, and the toxicity of pyrethroids to insects is attributable to their fast cuticular penetration and ‘knock down’ effect [16]. Indoxacarb (the second active ingredient present in the combination) enters the insect primarily through ingestion although it can be absorbed, to a lesser degree, through the insect cuticle. In vitro data show that indoxacarb is toxic to adult blowfly and mosquito larvae [17]. It is therefore likely that the sandfly feeding behavior only exposes the flies to the effects of topically applied permethrin, leading to minimal contribution of indoxacarb to the insecticidal activity.

The topical application of permethrin combined with indoxacarb (Activyl^®^ Tick Plus) was not associated with any local or systemic tolerance issue, supporting the safety profile of this product when used at the recommended dose.

## Conclusions

Activyl^®^ Tick Plus (indoxacarb and permethrin) applied topically to dogs at the minimum recommended dose had excellent anti-feeding efficacy against sandflies from 2 to 14 days post treatment with continuing significant effect to 29 days post-treatment. This treatment was well tolerated.

## Competing interests

The authors are employees of Merck/MSD Animal Health.

## Authors’ contributions

RF developed the study protocol, monitored the animal phase and contributed to interpretation of the results. AFS supported RF for these steps and assisted in the manuscript preparation. GMH performed the statistical analysis. All authors approved the completed manuscript.
